# The Story of the Hospitals

**Published:** 1886-10-02

**Authors:** 


### The Story of the Hospitals.

**Introduction.** In the "Story of the Hospitals," which I commence to-day, there lies before me a field almost unlimited in extent, a field in which one may garner with profit and with pleasure. Many persons have still an inborn horror, an irrational detestation of hospitals,—a horror, I need scarcely say, begotten of a superstitious ignorance. The sombrous tone of exterior walls is to them ample confirmation of the pictures they have formed in their wayward fancy. If among my readers there are some who may never have crossed the threshold of an hospital, and who still share the belief that these institutions are dread asylums of concentrated suffering, a practice-ground for students with the knife and the scalpel, I hope the light which I shall let in upon my story will dissipate the last vestiges of such lingering darkness. Whatever reproach our hospitals merited in a less gentle age has now abundantly been swept away. Those two great motors of life—light and love—are there. The wards are bright and cheery, often adorned with pretty pictures, mottoes, and beautiful flowers. By night and by day there are loving hands ever ready to smooth the pillow of the sufferer, while the resources of science are ransacked to the fullest to mitigate pain. But for these houses of mercy, how much more terrible would be the suffering among the poor, how much more heavy would be the death-roll of the Great City? As a nation we take a conscious pride in our hospitals, and justly so. The more intimate our acquaintance with the noble work they perform, the prouder we are of them. It is to increase our knowledge of these institutions, to present, as it were, to the reader a reflex of their daily life, that I have set myself to pen the "Story of the Hospitals." If I succeed in this, my object is attained.

**The East End.** I am going to ask my readers to accompany me to the Far East—I mean, of course, of London. This labyrinthine district of Whitechapel, Mile End, and Stepney is to the majority of West Londoners a *terra incognita.* Save those whom duty calls thither, or on those rare occasions when royalty pays a hurried visit, West London rarely sees East London. From the days when the Romans buried their urns and lachrymatories beneath Goodman's Fields, the district has had an unattractive history; like Mark Twain's jumping frog, it possessed no "particular point." Dickens, with the eye of the novelist, explored the purlieus and slums of the East-end, and to his graphic pen we stand largely indebted for our knowledge of "the life down East." The East-end of the town is a benighted region, densely populated, and to-day owns but the very scantiest attractions. The denizens, some million and a half in number, who are doomed to dwell to the east of Aldgate, are, for the most part, plodding and industrious workers, accustomed to participate in but very few of life's enjoyments. From day to day they spin out a bare existence, reconciled apparently to the hard and bitter lot which is their portion. The gardens and meadows, garden grounds and rope-walks, which existed here a century ago, have now given place to a mighty maze of bricks and mortar, intersected by the Whitechapel Road, a wide and commodious thoroughfare, once the great highway to Essex. It is to this spot that I have invited my reader mentally to accompany me. I will suppose him to have booked to that most dismal of all dismal stations on the District line—St. Mary's, Whitechapel. On emerging from the station, we turn to the right and walk a little way down the Whitechapel Road till we reach the first of those noble institutions which I have made it my mission to explore.

**The London Hospital.** We have come upon a plain brick building consisting of one extended front, and covering a site considerably over an acre in extent. The lodge-porter demands our business; we satisfy him, and enter; we are in the London Hospital.

**Its Early History.** As the boy said, who always "got up" the first chapter of Genesis for his Sunday school teacher, "there's nothing like beginning at the beginning." Let us follow for the nonce this instructive reasoning. The London Hospital, like many another great concern, had a very small commencement. Its inception was due to certain of the "faithful subjects" of King George II, who, "deeply affected with the distresses of their fellow creatures, and desirous to relieve some at least from perishing for want of proper care and assistance . . . did, on the third day of November 1740, form themselves into a society now commonly called 'The London Hospital'." These charitable men, whose primary object was to relieve "artificers, manufacturers, seamen in the merchant service, their wives and children, labouring under malignant and painful diseases and a variety of accidents," procured first of all a large house in Prescott Street, Goodman's Fields, where, in 1740, they opened the "London Dispensary." The old mansion really comprised four houses, and afforded space for about one hundred and thirty-six beds. The disrepair of the houses rendered them a source of constant trouble to the "Governors," who, in consequence, opened a subscription "to purchase a piece of ground and build a house more proper for enlarging and perpetuating their benevolent designs." The site they obtained—the "Mount"—was that on which the present Hospital stands. Money came in tolerably rapidly, and the Governors were, before long, able to put their wishes into a practical shape. On Oct. 15, 1752, Admiral Sir Peter Warren, K.B., laid the foundation stone of the new building, which was finished by December 1759. The new Hospital was fitted with about the same complement of beds as the old house in Prescott Street, but funds running short, eleven of the wards had for some time to remain unoccupied. A Charter of Incorporation was granted to the Governors by King George II on Dec. 9, 1758.

Troublous Days. The early years of the London Hospital were years of great pecuniary difficulty. Again and again the Governors made special appeals to a generous public, and by this means alone the doors of the institution were kept open. In 1807, in response to a gigantic effort, a collection of £26,349 was made, which enabled the Governors to carry out several pressing sanitary improvements, and to increase the in-patients to one hundred and eighty-seven. But even at this early period the demands on the Hospital were far in excess of its resources. The report for 1810, for instance, states:—"It possesses ample room for the accommodation of at least one hundred and fifty more patients, and every week the feelings of the Committee are distressed by numerous applicants whom they are under the painful necessity of rejecting because the funds of the charity are still inadequate to their maintenance." The war which was then raging on the Continent ran up the prices of provisions, and the Hospital was more than once on the point of being shut up. Again another appeal was made, and with success, Mr. Samuel Whitbread heading the list with a subscription of £1,000. The West Wing was then built, and the beds were increased from two hundred and sixteen to two hundred and seventy.

(*To be continued.*)

